# Supplementary material for: Bayesian refinement of protein structures and ensembles against SAXS data using molecular dynamics
Source: PLoS Comput Biol. 2017 Oct 18;13(10):e1005800. doi: 10.1371/journal.pcbi.1005800 (PMC5662244; doi:10.1371/journal.pcbi.1005800)
Supplement: S1 Archive — (BZ2) [file pcbi.1005800.s011.tar.bz2 › gromacs_4.62_bayesian_saxs_refi/share/html/online/trj.html]

trj file format


## trj file format

|  |  |
| --- | --- |
| Main Table of Contents | **VERSION 4.6** |
| GROMACS homepage | **Sat 19 Jan 2013** |

---

### Description

Files with the trj file extension contain the trajectory of a simulation.
In this file all the coordinates, velocities, forces and energies are
printed as you told GROMACS in your mdp file. This file is in binary
format an can be read with gmxdump.

```
% gmxdump -f traj.trj
```

or if you're not such a fast reader:

```
% gmxdump -f traj.trj | more
```

You can also get a quick look in the contents of the file (number of
frames etc.) using:

```
% gmxcheck -f traj.trj
```

---

http://www.gromacs.org
